# The neXtProt knowledgebase in 2020: data, tools and usability improvements

**DOI:** 10.1093/nar/gkz995

**Published:** 2019-11-14

**Authors:** Monique Zahn-Zabal, Pierre-André Michel, Alain Gateau, Frédéric Nikitin, Mathieu Schaeffer, Estelle Audot, Pascale Gaudet, Paula D Duek, Daniel Teixeira, Valentine Rech de Laval, Kasun Samarasinghe, Amos Bairoch, Lydie Lane

**Affiliations:** 1 CALIPHO group, SIB Swiss Institute of Bioinformatics, Geneva, Switzerland; 2 Department of microbiology and molecular medicine, Faculty of Medicine, University of Geneva, Geneva, Switzerland; 3 Haute école spécialisée de Suisse occidentale, Haute Ecole de Gestion de Genève, Carouge, Switzerland

## Abstract

The neXtProt knowledgebase (https://www.nextprot.org) is an integrative resource providing both data on human protein and the tools to explore these. In order to provide comprehensive and up-to-date data, we evaluate and add new data sets. We describe the incorporation of three new data sets that provide expression, function, protein-protein binary interaction, post-translational modifications (PTM) and variant information. New SPARQL query examples illustrating uses of the new data were added. neXtProt has continued to develop tools for proteomics. We have improved the peptide uniqueness checker and have implemented a new protein digestion tool. Together, these tools make it possible to determine which proteases can be used to identify trypsin-resistant proteins by mass spectrometry. In terms of usability, we have finished revamping our web interface and completely rewritten our API. Our SPARQL endpoint now supports federated queries. All the neXtProt data are available via our user interface, API, SPARQL endpoint and FTP site, including the new PEFF 1.0 format files. Finally, the data on our FTP site is now CC BY 4.0 to promote its reuse.

## INTRODUCTION

Comprehensive, current, high quality data, as well as innovative and powerful tools are necessary for researchers to make the most of the ever-increasing data relevant to human biology. neXtProt (1), a knowledgebase focusing exclusively on human proteins, leverages the expert manual annotation carried out at specialist resources and in-house to provide a single point of reference. Information concerning human protein function, cellular localization, tissular expression, interactions, variants and their phenotypic effect, post-translational modifications (PTMs), as well as peptide identified in mass spectrometry experiments and epitopes recognized by antibodies have been integrated from a number of resources. By doing so, neXtProt extends the contents of UniProtKB/Swiss-Prot (2) to provide a more comprehensive data set.

However, data alone is not sufficient for scientists to comprehend complex information rapidly. For this reason, neXtProt organizes the information concerning an entry in several views, with interactive viewers that allow the user to select the data displayed. We also provide tools to analyze and explore the data. A basic, full text search, as well as an advanced, SPARQL-based search, allow users to search the data in neXtProt. Additional tools have been implemented. Users can store and compare private lists of entries. The peptide uniqueness checker (3) determines which peptides are unambiguous and can thus be used to confidently identify protein entries (4).

In this manuscript, we describe the latest progress on developing neXtProt. Since 2016, three major data sets have been integrated. Firstly, high quality, tissular expression data from the Human Protein Atlas (HPA) obtained by RNA-seq (5) has been added. Secondly, information annotated from the literature on the function, cellular localization, interactions and phosphorylations carried out by human protein kinases has been incorporated. Lastly, variant frequency data from the Genome Aggregation Database (gnomAD) (6) extends the information on sequence variations at the protein level. We also report on improvements made to the peptide uniqueness checker and the implementation of the new protein digestion tool. Finally, we present improvements to the web site and SPARQL endpoint to improve the accessibility and usability of the neXtProt data.

### neXtProt data overview

The first neXtProt release in April 2011 contained data from UniProtKB, Ensembl, HPA, Bgee and GOA. Since then neXtProt has been steadily incorporating new data from additional resources, with a particular emphasis on expression data, proteomics data and variant data. The current neXtProt release was built using human genome assembly GRCh38 (7). The data from UniProtKB (2) is currently supplemented with data from Bgee (8), HPA (5,9), PeptideAtlas (10), SRMAtlas (11), GOA (12), dbSNP (13), Ensembl (14), COSMIC (15), DKF GFP-cDNA localization (16,17), Weizmann Institute of Science's Kahn Dynamic Proteomics Database (18), IntAct (19), GlyConnect (20), gnomAD (6), as well as in-house curated data (21,22). Table 1 summarizes the changes in the content since our last neXtProt update (1).

**Table 1.** Data content of neXtProt data release 2019-08-22

Entries	Statistics	Change since data release 2016-08-25	Source(s)
Entries	20 399	+338	UniProtKB
Isoforms (Sequences)	42 410	+386	UniProtKB
Binary interactions	240 010	+99 740	IntAct, neXtProt
Post-translational modifications (PTMs)	190 921	+48 468	UniProtKB, neXtProt, PeptideAtlas, GlyConnect
Variants (including disease mutations)	6 019 871	+1 075 957	UniProtKB, COSMIC, dbSNP, neXtProt, gnomAD
Phenotypic annotations	19 602	+11 588	neXtProt
Entries with a molecular function	17 177	+654	GOA, neXtProt
Entries with a biological process	16 964	+692	GOA, neXtProt
Entries with an expression profile	19 367	+1038	Bgee, HPA, neXtProt
Entries with a disease	4553	+637	UniProtKB
Entries with proteomics data	18 727	+1448	PeptideAtlas, neXtProt
Entries with an experimental 3D structure	6 505	+765	PDB via UniProtKB
Cited publications	115 935	+16 013	All resources

The data in the UniProtKB/Swiss-Prot (Reviewed) entries for Homo sapiens (TaxID: 9606) having the keyword Complete proteome (KW-0181) provide the groundwork for neXtProt. In order to evaluate the improvement in coverage through the integration of data from sources other than UniProtKB, we determined the number of entries in neXtProt with data from UniProtKB with that having data from any source using SPARQL queries (Table 2). UniProtKB provides excellent coverage for a single resource; it thus provides a good foundation for the construction of neXtProt. The incorporation of data from additional sources considerably improves the coverage—over 78% of entries in neXtProt have information about the function, cellular localization, interactions, expression, post-translational modifications and variants.

**Table 2.** Coverage in neXtProt data release 2019-08-22

Annotations	Entries with evidence from UniProtKB (%^a^)	Entries with evidence from any source (%^a^)
GO molecular function	12 360 (60%)	17 177 (84%)
GO biological process	11 665 (57%)	16 964 (83%)
GO cellular component	14 581 (71%)	18 129 (89%)
Subcellular location	16 590 (81%)	18 527 (91%)
Binary interactions	8653 (42%)	16 411 (80%)
Expression	9876 (48%)	19 527 (96%)
Peptide mapping	0 (0%)	18 727 (92%)
Antibody mapping	0 (0%)	16 423 (80%)
Post-translational modifications (PTMs)	14 000 (69%)	15 905 (78%)
Variants	12 919 (63%)	19 621 (96%)

^a^The total number of entries is 20 399.

### RNA-seq

**Figure 1.**
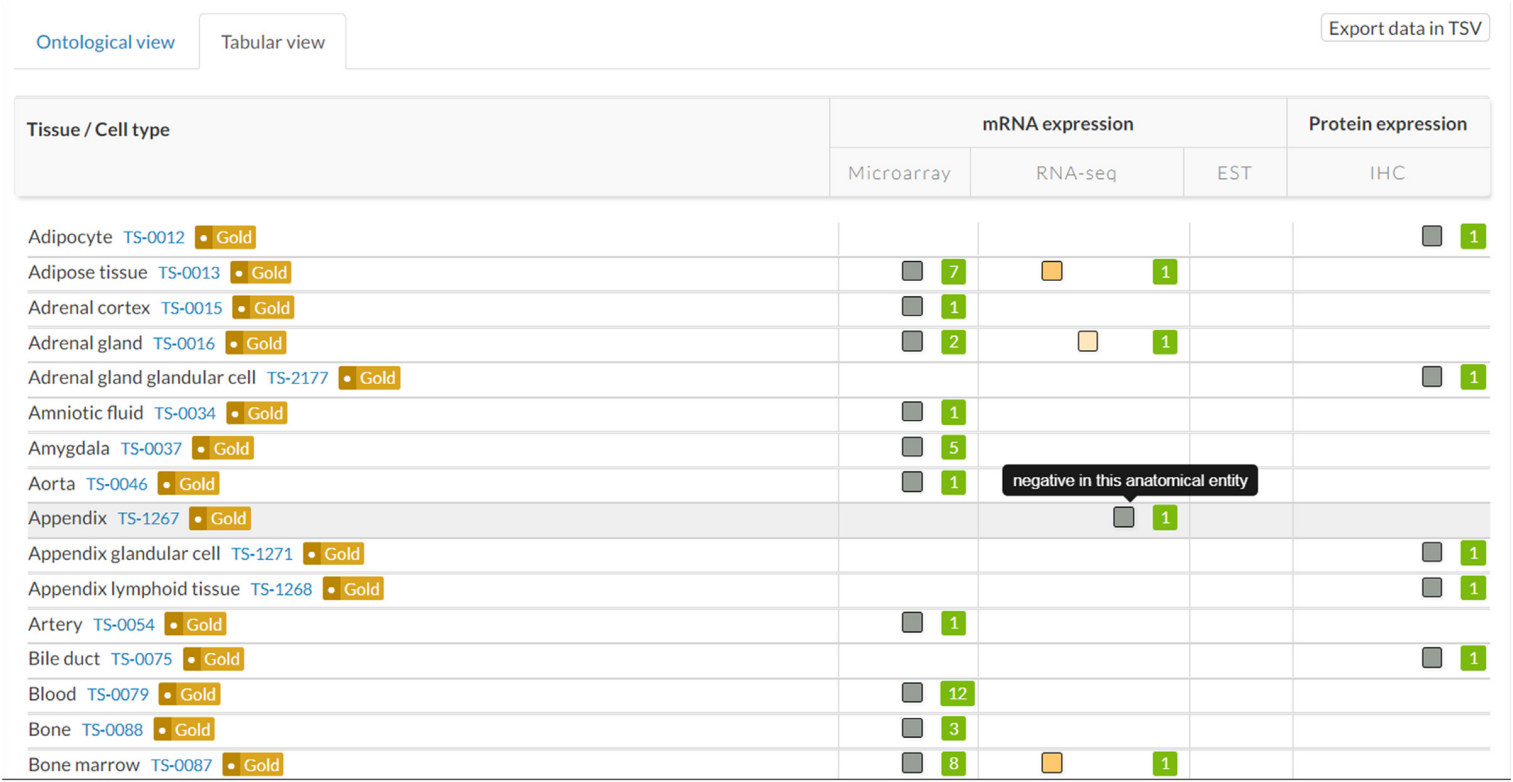
Tabular view showing the expression data for insulin (NX_P01308). Expression data at the mRNA and protein level are displayed in the same semi-quantitative manner for easier comparison. Four levels of expression (undetected, low, medium, high) are possible. Mousing-over the data point displays the expression level textually.

We incorporated the RNA-seq data from 37 different normal tissues from Human Protein Atlas. As RNA-seq data is highly accurate for quantifying expression levels with high reproducibility, this improved the expression data provided in neXtProt at the level of the transcript, which until then came from microarray and expressed sequence tag (EST) data. While RNA-seq is quantitative, the semi-quantitative expression values (undetected, low, medium and high) provided by HPA were taken and are displayed in the same manner as the other data in the *Expression view* to make for easier comparison (Figure 1). This also enables the RNA-seq data to be queried in the same manner using SPARQL.

### Protein kinases

Another new large set that was integrated is a set of manual annotations that we have created to capture a wide range of published experimental results concerning 300 protein kinases. The proteins phosphorylated by these kinases, as well as whenever possible the specific amino acid residue which is phosphorylated, were annotated. This phosphorylation data set complements that of PeptideAtlas, which only provides phosphorylation sites. With this data, neXtProt now contains 10 725 entries (52%) with a phosphorylation on a serine, threonine or tyrosine residue. Of the 115 822 phosphorylation sites, only 5219 are associated with a specific protein kinase. The substrates of a specific protein kinase can be retrieved using a SPARQL query; for instance, the query NXQ_00069 retrieves all proteins phosphorylated by SYK. The protein's molecular function and its involvement in a biological process were captured using Gene Ontology (GO) terms (23,24). Binary interactions with human proteins were also annotated and are displayed in the *Interactions view*.

As with the phenotypic data described in our previous report (1), this protein kinase data is available in the new Protein kinase function portal. Accessible from the top menu ‘Portals’, the data are presented in tabular form, with each column being searchable and sortable. More details concerning the experimental context for the data in all portals is now provided. Two new columns, labeled Cell line / Tissue and Experimental details, can be used to filter the data. The data in the portals can be downloaded in CSV format, copied or printed. The entry accession (AC) corresponding to the annotation subject has also been added.

### Variant frequency

To date the corpus of variant data in neXtProt covers variants observed in health and disease, as well as the phenotypic effect of the variants. The neXtProt database contains over six million single amino acid variations imported from UniProtKB, dbSNP, COSMIC and manually annotated from the literature, but it is difficult to make use of this variant data in the absence of information about their frequencies in human populations. The Genome Aggregation Database (gnomAD) (6) spans 126 216 exome sequences and 15 136 whole-genome sequences extracted from a variety of large-scale sequencing projects and provides computed allele frequencies for most of the reported variants. We have thus integrated variant frequency information from the gnomAD version 2.1.1.

**Figure 2.**
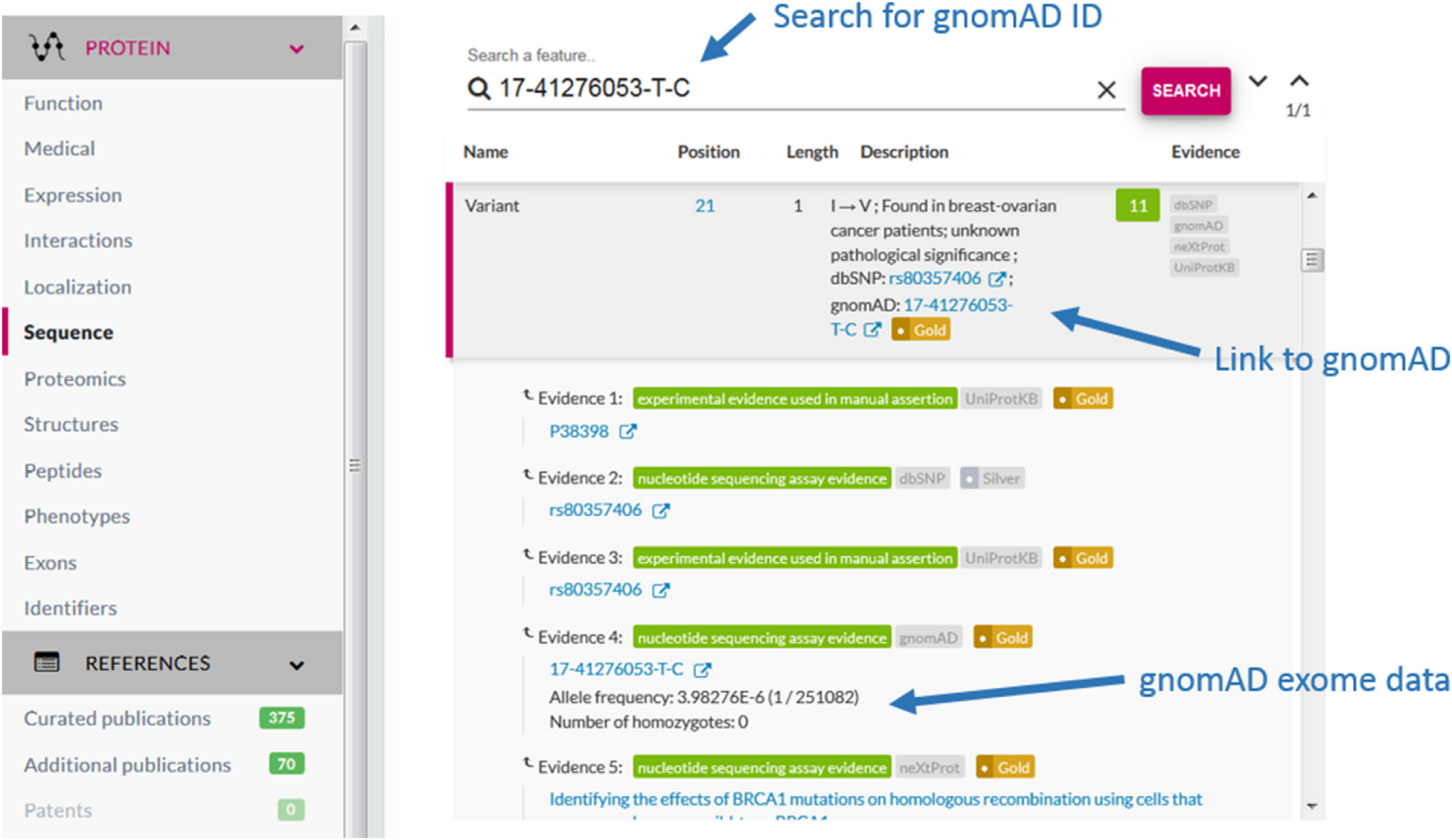
Allele frequency information in the *Sequence view* for BRCA1 (NX_P38398) variants. To find a gnomAD variant, search in the feature table with the gnomAD ID. A link to the corresponding variant in gnomAD is found in the description of the variant and the evidence. The allele frequency, with the allele count and allele number in brackets, as well as the homozygote count, are displayed in the evidence.

neXtProt now contains 18 685 entries (92%) and 2 691 323 variants (45%) with frequency data from gnomAD. We display the number of times the allele was sequenced (allele count), the number of individuals homozygous for the allele (homozygous count), the total number of alleles sequenced (allele number) and the allele frequency in the evidence (Figure 2). SPARQL queries can be used to answer questions such as which variants have a frequency greater than 0.1 (NXQ_00255) or which variants are frequently found in a homozygous state (NXQ_00256).

### Peptide uniqueness checker

The peptide uniqueness checker (3) allows scientists to define which peptides can be used to validate the existence of human proteins by determining whether a peptide maps uniquely versus multiply to human protein sequences taking into account isobaric substitutions, alternative splicing and single amino acid variants. It was adapted to take into account the entries with identical isoform sequences. Peptides matching such sequences were considered to be ‘Found in other entries’; such peptides are now considered ‘Pseudo-unique’ and entries or isoforms having identical sequences are labelled with an asterisk in the results. We also added a brief description of what the tool does and examples covering all cases in the input section of the interface. In response to user requests, we added in the display of the results icons so as to help color-blind users to distinguish whether peptides are ‘unique’, ‘pseudo-unique’ or ‘not unique’.

### Protein digestion

**Figure 3.**
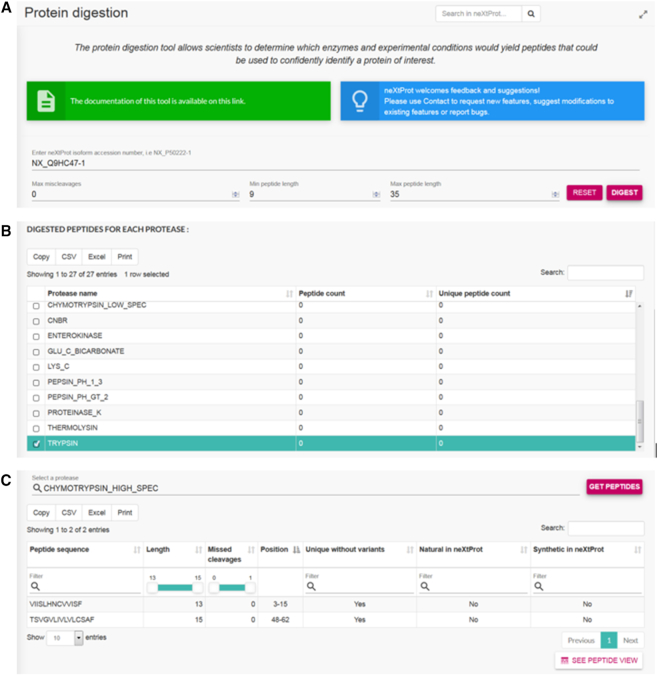
Protein digestion tool. (**A**) Input form requiring the neXtProt isoform accession number for the protein to be digested. Default digestion parameters (maximum number of miscleavages, minimum peptide length and maximum peptide length) can be modified by the user. (**B**) Peptide count and unique peptide count for the digestion with 27 proteases or conditions. Select a protease to see the peptides obtained. (**C**) Table displaying information about the peptides obtained with the selected digestion conditions. The peptide sequence, length, number of missed cleavages, position in the sequence, whether the peptide is unique or not (without taking into account variants) and whether the peptide is found in neXtProt, as a natural and synthetic (SRM peptide) are displayed. A link to the neXtProt *Peptide view* of the entry is provided.

A new tool that performs *in silico* protein digestion is now available at https://www.nextprot.org/tools/protein-digestion. This tool allows the user to input the neXtProt accession for a specific isoform of an entry and returns the peptide sequences obtained upon digestion. Combined with the peptide uniqueness checker, it can be used to determine which proteases can be used to identify trypsin-resistant proteins by mass spectrometry. For example, trypsin digestion of the cancer-testis antigen CTAGE1 isoform 1 (NX_Q9HC47-1) does not yield a peptide of 9–35 aa (Figure 3A and B); however digestion with high specificity cleavage with chymotrypsin (CHYMOTRYPSIN_HIGH_SPEC) results in two, non-overlapping proteotypic peptides (Figure 3C).

### Web site and API

In our last update (1), we reported changes in the home page, the navigation and the documentation. Since then, we have completely revamped the neXtProt website. All entry, publication and controlled vocabulary term pages have been rewritten so that they load faster, thereby improving their usability. This was necessary, as the amount of data in neXtProt has increased considerably over the years. We also introduced a Tabular view in the *Expression view* (Figure 1). This new view shows the expression level for every tissue assayed. An export functionality allows all the experimental expression data for the entry to be downloaded in tab-delimited format. We also combined the Gene Identifiers and Protein Identifiers entry views in a single view. This allows users to find all the identifiers for an entry in the *Identifiers view*. Having completed the revamping of our web site, our original website and the associated API were disconnected. Users should now use the API at https://api.nextprot.org/.

### Querying using SPARQL

In the last four years, neXtProt has been promoting the use of SPARQL, a semantic query language for databases, to explore human data. Semantic technologies can help to generate innovative hypotheses where classical data mining tools have failed (protein function prediction, drug repositioning, etc.). neXtProt provides over 160 pre-built queries in its Advanced search (https://www.nextprot.org/proteins/search?mode=advanced) and SNORQL (https://snorql.nextprot.org/) interfaces. The former retrieves entries, while the latter retrieves any data, meeting the defined criteria. The queries illustrate the types of questions that can be answered using SPARQL. The data model documentation is provided in the SNORQL Help and a user guide to help the user in his/her first steps in SPARQL has been published (25).

**Figure 4.**
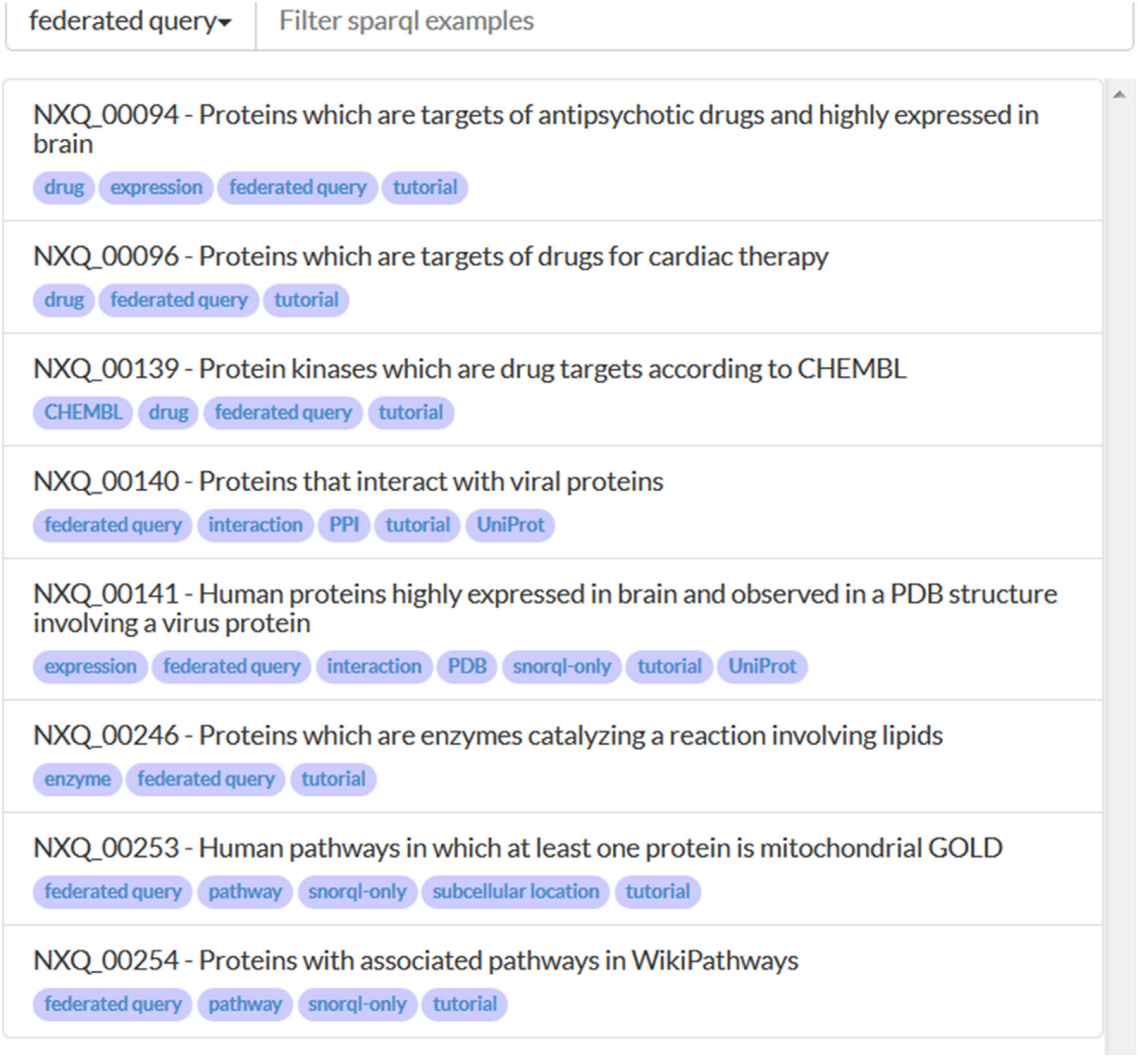
Federated SPARQL query examples. Screenshot showing all queries tagged ‘federated’ in the neXtProt SNORQL interface.

The use of SPARQL allows users to run federated queries across multiple resources relevant to human biology. Thus SPARQL allows queries to be carried out on data both in and beyond neXtProt. Figure 4 shows examples of federated queries. Currently these also query data in ChEMBL, DrugBank, PDB, Rhea, UniProtKB and WikiPathways. The neXtProt SPARQL endpoint (https://api.nextprot.org/sparql) also supports federated queries.

### Data availability

High-throughput identification and quantification of proteins, including sequence variants and post-translational modifications (PTMs) in biological samples by mass spectrometry-based proteomics is becoming commonplace. While sequence variations need to be taken into account in the search space used to analyze the data, doing so remains a challenge. The Proteomics Standards Initiative (PSI) has designed and implemented the PSI extended FASTA format (PEFF) (26) to facilitate the search for known sequence variants and PTMs. Based on the FASTA format, PEFF encodes substantially more metadata about the sequence collection as well as individual entries, including support for encoding known sequence variants, PTMs, and proteoforms. We have worked closely with PSI with the outcome that neXtProt was the first resource to implement the PEFF v1.0 format. This ensures the interoperability of neXtProt data with the sequence search engine Comet. We expect that the PEFF format will soon be adopted by other MS software tools.

All the neXtProt data, dating back to the first release in 2011, can be downloaded. In order to foster the reuse of the data in neXtProt, we have lifted the ‘no derivatives’ restriction applying to the data available from our FTP site (ftp://ftp.nextprot.org/pub/). As of 21 February 2018, the license applying to the use of our data available is CC BY 4.0.

## CONCLUSION

The past years have seen numerous changes in neXtProt. We have incorporated new, high quality RNA-seq expression, protein kinase function and variant frequency data, and updated the data from practically all our sources, in order to provide an up-to-date, comprehensive data set. Some changes, such as the rewriting of our user interface, have been necessary to cope with the increase in data. Others, such as the modification to the peptide uniqueness checker, were prompted by changes in the data and feedback from users. We continue to support research in proteomics and have implemented the protein digestion tool to enable researchers to find alternatives to trypsin when planning experiments to validate the existence of a protein. We welcome feedback on our data, tools and website and encourage users to contact us using the e-mail support@nextprot.org, Twitter (@neXtProt_news) or ResearchGate (neXtProt project).
